# A hypermobile prophage in the genome of a key human gut bacterium

**DOI:** 10.1371/journal.pbio.3003071

**Published:** 2025-04-01

**Authors:** Andrey N. Shkoporov, Colin Hill

**Affiliations:** 1 School of Microbiology & APC Microbiome Ireland, University College Cork, Cork, Ireland; 2 Department of Medicine, University College Cork, Cork, Ireland

## Abstract

Phages infecting anaerobic bacteria are highly abundant in the mammalian gut, but their biology and ecological impact are poorly understood. A new PLOS Biology study provides a glimpse into the disruptive biology of the Hankyphages, parasites of the ubiquitous *Bacteroidaceae*.

The *Bacteroidaceae* (including genera *Bacteroides* and *Phocaeicola*) is one of the most abundant families of the mammalian gut microbiome [1]. High genomic plasticity and functional adaptability is a vital attribute if these bacteria are to thrive in the gut, where they occupy an important niche as “primary degraders” [2]. *Bacteroidaceae* utilize their numerous and diverse polysaccharide utilization loci (PULs) to break down complex dietary glycans [3]. The ability of *Bacteroidaceae* bacteria to quickly switch on and off the expression of individual PULs and various surface structures is attributed to regulatory systems based on site-specific DNA recombination (phase variation) and transcriptional antitermination [4,5].

Unsurprisingly, the ecological success and subsequent abundance of *Bacteroidaceae* in the gut attracts attention from a variety of bacteriophage parasites. *Crassvirales*, Gubaphages, Hankyphages, “Quimbyviridae”, and “Flandersviridae” among many others, piggyback on these successful gut symbionts by infecting and causing lysis (lytic infection) or inserting themselves into bacterial genomes (lysogenic infection). These viruses infecting *Bacteroidaceae* are among the most abundant in the human gut [6,7]. Selective pressure exerted by lytic phages necessitates constant adaptations in *Bacteroidaceae* bacteria, including shuffling of interchangeable protective capsules and S-layer proteins [5], to limit the likelihood of population extinction. Insertion of temperate phages can directly lead to alteration of host gene expression and physiology. For example, upon integration, *Phocaeicola* prophage BV01 disrupts an important regulatory pathway, leading to a reduction in bile salt hydrolase expression [8]. It is almost certain that there are numerous other as yet undiscovered mechanisms through which *Bacteroidaceae* phages affect the molecular physiology of their hosts, modulate ecological networks in the gut, and influence the human host.

The recent PLOS Biology study by Vendrell-Fernández et al. [9] in PLOS Biology investigates a previously uncultured phylogenetic group of phages named Hankyphages and provides some important insights into their unusual biology. First discovered in 2018, these viruses attracted attention for being widespread and active in the human gut [10]. Hankyphages carry diversity-generating retroelements that enable rapid mutation of genes encoding receptor-binding proteins (see Fig 1), a strategy that is likely responsible for expanding their host range to at least 13 different species of *Bacteroides and Phocaeicola*. This study [9] focused on a particular variant of Hankyphage located in the genome of *Bacteroides thetaiotaomicron*, a well characterized and genetically accessible species that serves as a perfect platform for molecular studies.

**Fig 1:**
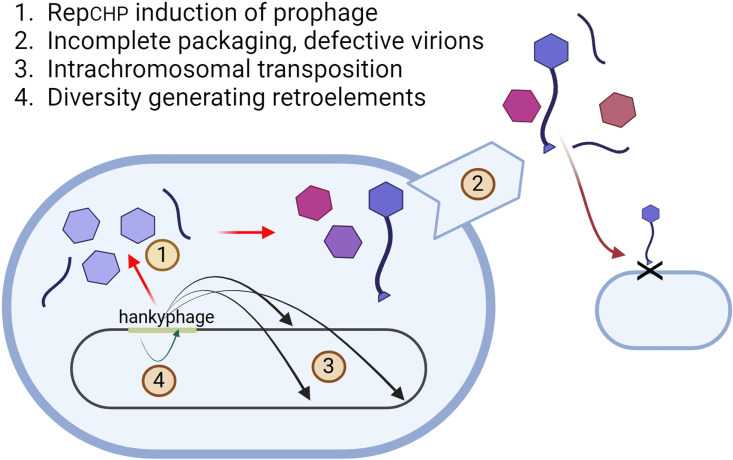
Mechanisms of Hankyphage infection of *Bacteroidaceae.* Hankyphage has multiple mechanisms of generating randomness in its own and its bacterial host genomes. (1) Rep_CHP_-dependent spontaneous induction of prophage, (2) incomplete packaging leading to defective virions and free tails in combination with low-level complete packaging and potential transduction events, (3) intrachromosomal transposition leading to genetic variation in the host genome, and (4) diversity generating retroelements within the phage genome impacting the host range of the phage. Created with BioRender.

Interestingly, this Hankyphage maintains a constant level of spontaneous induction in overnight cultures, generating 10^6^–10^8^ virions per mL. Paradoxically, the phage is unresponsive to common prophage-inducing chemical and physical stimuli. The CI repressor gene that is responsible for prophage silencing is nevertheless active, as was demonstrated by a series of elegant CRISPRi experiments. Whether spontaneous induction also occurs in the gut or is simply a result of growth in the lab in pure cultures remains to be established.

Phage generated through spontaneous induction did not produce lytic infections in Hankyphage-naïve isolates, or in a prophage cured variant of the parental strain. Similarly, no horizontal transfer of the prophage to other strains was observed. In part, this was explained by detailed microscopic and biochemical evidence indicating the formation of predominantly defective virions with incomplete DNA packaging, at least under the conditions studied. However, it remains a mystery why the small fraction of apparently complete virions carrying full-sized genomes also failed to produce lytic infections or lysogenise isogenic bacterial hosts. It may be that this phage is only capable of correct assembly and packaging under very specific conditions in the gut, similar to what was observed for phage BV01 [8].

Hankyphage also uses a somewhat unusual mechanism of replication by transposition. The prophage integration sites are random and will vary in different clonal lineages. This can result in different gene disruptions and/or polar effects on transcription of genes adjacent to the integration site, potentially leading to significant phenotypic diversity within the mixed population of lysogens. To our knowledge, this mechanism has never been previously described in the phylum Bacteroidota and deserves further investigation, both in terms of the replication mechanism itself and on the impact of random Hankyphage insertions on fitness and adaptability of its *Bacteroidaceae* hosts.

Another important aspect of replication by transposition is the potential ability of Hankyphages to carry out generalized transduction. Every completely packaged Hankyphage contains a random 1–2.3 kb fragment of bacterial DNA attached to its 3′ end. Even where superinfection inhibition applies this could result in successful homologous recombination of the transduced portion of bacterial DNA with the recipient genome. Given the known plasticity and intraspecies diversity of *Bacteroidaceae* genomes, this could have massive implications for the evolution of these bacteria in the gut. The role of phages as genetic couriers in the microbiome is only starting to emerge [11], a process potentially enabling access of related bacterial strains to the entire species pangenome. The discovery of possible transducing phages in a major gut symbiont can accelerate progress in this field.

The study by Vendrell-Fernández et al. [9] provides an intriguing glimpse into the complex biology of the transposing Hankyphages. While their findings are important and insightful, many puzzles remain. Does complete Hankyphage assembly only occur under very specific gut conditions? How does Hankyphage transposition affect population structure and fitness of its host? Is Hankyphage capable of generalized transduction and what effect would it have on the evolution and ecological specialization of *Bacterodaceae*? Studies such as this one reinforce our conviction that we will never fully appreciate the complexity and functionality of the gut bacteriome without factoring in the roles of the many ‘partner’ phages present in this complex ecosystem.
